# Comparative genomic analysis of Clostridioides difficile isolates from symptomatic and asymptomatic paediatric patients

**DOI:** 10.1099/mgen.0.001610

**Published:** 2026-02-02

**Authors:** Lewen Tu, Runjie Wang, Gaojie Liu, Rulan Liu, Yongchao Mei, Xufei Wang, Lin Ye, Fangfei Xiao, Lu Wang, Guangjun Yu, Yizhong Wang, Xiangna Niu, Ting Zhang, Xiaolu Li

**Affiliations:** 1 Department of Gastroenterology, Hepatology and Nutrition, Shanghai Children’s Hospital, School of Medicine, Shanghai Jiao Tong University, Shanghai, PR China; 2 Gut Microbiota and Metabolic Research Center, Institute of Pediatric Infection, Immunity and Critical Care Medicine, School of Medicine, Shanghai Jiao Tong University, Shanghai, PR China; 3 The Second Affiliated Hospital, School of Medicine, The Chinese University of Hong Kong, Shenzhen & Longgang District People’s Hospital of Shenzhen, Shenzhen, PR China; 4 Shanghai Engineering Research Center for Big Data in Pediatric Precision Medicine, Shanghai, PR China; 5 Shanghai Winnerbio Technology Co., Ltd., Shanghai, PR China

**Keywords:** asymptomatic carrier, children, *Clostridioides difficile*, symptomatic infection, whole-genome sequencing

## Abstract

*Clostridioides difficile* infection (CDI) imposes a substantial clinical burden in paediatric populations. However, the high prevalence of asymptomatic colonization, especially in children under 2 years of age, complicates the distinction between true infection and non-pathogenic carriage. This diagnostic uncertainty hinders appropriate treatment decisions and complicates infection prevention efforts. A 6-year retrospective cohort study was performed at Shanghai Children’s Hospital to characterize the epidemiology and clinical features of CDI and asymptomatic colonization in paediatric patients. Stool specimens were cultured for *C. difficile*, and isolates underwent whole-genome sequencing to perform multilocus sequence typing, identify SNPs and characterize functional gene content via Clusters of Orthologous Groups (COG) analysis. Mutations in genes associated with toxin production were analysed to assess genetic differences between clinical isolates from infected patients and asymptomatic carriers. In addition, comparative genomic analysis was performed to assess variations in virulence-associated genes, antimicrobial resistance (AMR) genes and genes involved in quorum sensing (QS). Colonization factors (CFs) were also characterized to elucidate potential mechanisms differentiating asymptomatic colonization from symptomatic infection. And we conducted *in vitro* experiments on toxin B-variant strains. A total of 23 sequence types (STs) were identified among isolates from 39 asymptomatic carriers and 61 symptomatic patients, with greater ST diversity observed in the infection group compared to the colonization group. COG analysis demonstrated an increased representation of uncharacterized functional categories in the infection group, suggesting a potential role for novel genes in pathogenesis. Patterns of virulence factor presence, AMR genes and QS gene distribution were comparable between the two groups, as were mutations in toxin regulation genes. Notably, six isolates belonging to ST37 and ST81, characterized by the absence of *tcdA* and presence of *tcdB*, exhibited a high frequency of mutations. *In vitro* experiments demonstrated that these strains exhibited higher biofilm formation capacity and elevated transcriptional levels of both *tcdB* and *spoA*. Additionally, no significant differences were detected in the distribution of CFs. Our findings contribute to the growing understanding of the genomic determinants and their functional roles underlying paediatric CDI severity, providing more evidence for improved diagnostic and therapeutic strategies.

**Impact Statement**

*Clostridioides difficile* infection (CDI) poses significant diagnostic challenges in children due to high rates of asymptomatic colonization. This comprehensive genomic analysis of paediatric CDI provides critical insights into the molecular determinants that differentiate symptomatic infection from asymptomatic colonization. By employing whole-genome sequencing and functional genomic approaches, we identified key genetic variations, including novel uncharacterized functional categories and specific mutations in toxin B-variant strains (ST37 and ST81) that may contribute to pathogenesis. This work provides crucial insights into CDI pathogenesis mechanisms and establishes a genomic foundation for developing improved diagnostic approaches and targeted therapeutic strategies in paediatric healthcare settings.

## Data Summary

Raw sequencing data have been deposited in the NCBI database under study accession number PRJNA1047048. The accession numbers of strains in our study are provided in Table S5.

## Introduction

*Clostridioides difficile* is an anaerobic, spore-forming bacillus transmitted via the faecal-oral route and is recognized as a leading cause of antibiotic-associated diarrhoea worldwide [1,2]. Its key virulence factors (VFs) include toxins – TcdA (toxin A), TcdB (toxin B) and occasionally a *C. difficile* binary toxin (CDT) [3]. Clinically, *C. difficile* infection (CDI) manifests across a broad spectrum, from mild antibiotic-associated diarrhoea to severe fulminant colitis, which may lead to complications such as toxic megacolon [4].

Although *C. difficile* has the capacity to cause symptomatic infection, it can also persist as an asymptomatic colonizer within the gastrointestinal tract [5]. While CDI is well-characterized in adults, children exhibit unique epidemiological features and clinical manifestations [6,9]. Notably, a substantial proportion (~75%) of paediatric CDI cases are community-associated [10,11]. Crucially, age-dependent variation is observed in detection rates, with asymptomatic colonization rates surpassing 40% in infants under 1 year of age [6]. These findings suggest that infants, especially those colonized with toxigenic *C. difficile* strains, may serve as significant reservoirs for transmission within the community [7,8].

This high prevalence of asymptomatic carriage complicates the clinical distinction between true CDI requiring treatment and non-pathogenic colonization in children. Accurate differentiation is vital for guiding appropriate therapeutic decisions and infection control measures. Whole-genome sequencing (WGS) has substantially deepened our understanding of *C. difficile* genetic diversity, evolutionary history, epidemiological features and pathogenic mechanisms [12]. However, genomic studies focusing on asymptomatic *C. difficile* colonization – particularly in paediatric populations – remain limited. This significant knowledge gap constrains our understanding of the underlying genomic and mechanistic factors that differentiate harmless colonization from symptomatic disease in children.

To address this gap and elucidate genomic features distinguishing paediatric CDI from asymptomatic carriage, we conducted a comparative genomic analysis of 100 *C*. *difficile* isolates collected from paediatric patients. This cohort comprised both children with symptomatic CDI and asymptomatic carriers. We employed molecular epidemiological approaches and comparative genomics to characterize the strains and identify potential discriminators between infection and colonization states in this vulnerable population. Furthermore, we conducted some *in vitro* experiments on toxin B variants to explore their virulence characteristics.

## Methods

### Patients

This study was conducted between 2018 and 2024 at Shanghai Children’s Hospital, involving both outpatient clinics and inpatient wards within the Department of Gastroenterology. All enrolled patients were under 18 years of age.

Based on the 2017 clinical practice guidelines from the Infectious Diseases Society of America and the Society for Healthcare Epidemiology of America [13], clinical CDI in children is defined as three or more watery stools per day (Bristol type 6–7), along with a positive stool toxin test, detection of toxigenic *C. difficile* by PCR or evidence of pseudomembranous colitis on colonoscopy. Asymptomatic colonization is defined as a positive PCR (nucleic acid amplification test, NAAT) test in the absence of diarrhoea within the previous 48 h and no history of CDI treatment. These definitions were used to classify participants into symptomatic and asymptomatic groups in our study.

### Collection of clinical *C. difficile* isolates

In this study, symptomatic CDI was defined as a positive stool NAAT result accompanied by the presence of diarrhoea, whereas an asymptomatic carrier was defined as an individual with a positive stool NAAT result in the absence of diarrhoea. A total of 600 stool specimens that tested positive for NAAT were collected from paediatric patients. Samples were cultured on cycloserine–cefoxitin–fructose agar (Oxoid, UK) for selective isolation [14]. Plates were incubated under anaerobic conditions using an anaerobic chamber (ELECTROTEK AW500 SG, UK). Suspected *C. difficile* colonies were confirmed by PCR and Sanger sequencing targeting the 16S rRNA gene and the housekeeping gene *tpi*. For subsequent characterization, a representative, well-isolated colony with typical *C. difficile* morphology was selected and preserved from each positive plate.

### WGS and MLST typing of *C. difficile*

Genomic DNA was extracted from the bacterial pellet using the Wizard^®^ Genomic DNA Purification Kit (Promega, USA). High-quality genomic DNA (≥1 µg) was used to construct sequencing libraries with an average insert size of ~400 bp, using the TruePrep DNA Library Prep^™^ Kit V2 (Illumina, USA) for paired-end sequencing. WGS was carried out on the Illumina NovaSeq 6000 platform (Illumina, USA) using a paired-end sequencing strategy with a read length of 150 bases. As previously described [15], strain typing and clade assignment were performed by aligning the assembled genomic scaffolds to the PubMLST database, based on the sequences of seven housekeeping genes.

### Phylogenetic analysis of *C. difficile* strains and COG analysis of strains

A phylogenetic tree based on core SNPs was constructed using kSNP3 [16] with the maximum-likelihood (ML) method. SNP differences between isolates were identified using Snippy, and isolates with ≤2 core genome SNPs (cgSNPs) were defined as belonging to the same clonal group (CG), according to the criteria established by Zhou *et al.* [17]. Orthologous gene family analysis was performed using OrthoFinder v2.4.0 [18] with default parameters. Representative protein sequences from each orthologous gene family were functionally annotated and classified using the Clusters of Orthologous Groups (COG) database.

### Sequence analysis of toxin loci, PaLoc and CdtLoc

Amino acid sequence variations in the TcdA and TcdB proteins were analysed using a custom Python script for pairwise alignment. The distribution of mutations in TcdA and TcdB across all isolates was statistically assessed, and the results were visualized as a heatmap using the R programming language. In addition, linear comparisons of virulence gene clusters and pathogenicity islands were conducted using Easyfig v2.2.3 [19].

### Identification of quorum-sensing genes, virulence genes and antimicrobial resistance genes

Quorum-sensing (QS) genes were identified through blast alignment. The *agr* locus in *C. difficile* comprises four genes: *agrA*, *agrB*, *agrC* and *agrD*. These genes contain multiple variants, with *agrA* harbouring *agrA2*; *agrB* comprising *agrB1*, *agrB2* and *agrB3*; *agrC* containing *agrC2* and *agrC3*; and *agrD* comprising *agrD1*, *agrD2* and *agrD3*. Based on these variants, three distinct *agr* QS systems have been identified: *Agr1* (*agrB1*, *agrD1*), *Agr2* (*agrA2*, *agrC2*, *agrD2*, *agrB2*) and *Agr3* (*agrB3*, *agrD3*, *agrC3*) [20,21]. VFs were annotated using the VFDB database [22], and antimicrobial resistance genes (ARGs) were predicted using the CARD database [23]. Heatmaps illustrating the distribution of QS genes, VFs and ARGs across all isolates were generated using the R programming language.

### Colonization factor and colonization module analysis

Colonization factors (CFs) were identified using DIAMOND software [24] with the ‘-ultra-sensitive’ option, following the approach described by Liu *et al*. [25]. The alignment parameters were set as follows: ALIGNMENT_QUERY_COVERAGE=66 and ALIGNMENT_SUBJECT_COVERAGE=50. A heatmap illustrating the distribution of CFs across all isolates was generated using the R programming language.

### *In vitro* assays of *C. difficile*

For *in vitro* virulence phenotype analysis, we compared six clinical *C. difficile* isolates harbouring TcdB mutations with three randomly selected clinical *C. difficile* isolates with wild-type TcdB. The standard *C. difficile* strain ATCC 43255 was included as a control. The six TcdB mutant strains comprised two ST81 isolates and four ST37 isolates, while the three wild-type (non-mutant) TcdB strains were all ST54 isolates. The reference strain ATCC 43255 belongs to ST46.

### Biofilm formation and virulence gene expression

*C. difficile* strains were cultured in brain heart infusion (BHI) medium at 37 °C for 12 h. The overnight culture was then diluted 1:100 in BHISG medium (BHIS supplemented with 1.8% glucose) and thoroughly mixed by vortexing [26]. Aliquots of 1 ml diluted culture were dispensed into each well of a 24-well microplate and incubated at 37 °C for 3 days under anaerobic conditions to promote biofilm formation. The resulting biofilms were stained with 0.1% (w/v) crystal violet solution, followed by gentle washing with sterile PBS to remove excess stain. Finally, the bound dye was solubilized in 33.3% acetic acid, and biofilm formation was quantified by measuring absorbance at 570 nm using a microplate reader.

To investigate the expression of key virulence and regulatory genes, we performed quantitative real-time PCR (qRT-PCR) analysis. *C. difficile* strains were cultured in BHI medium at 37 °C for 12 h to reach the exponential growth phase. Total bacterial RNA was subsequently extracted using the Bacteria RNA Extraction Kit (Vazyme, Nanjing, China). The extracted RNA was then reverse transcribed using HiScript III RT SuperMix for qPCR (Vazyme, Nanjing, China). The relative expression levels of key toxin genes (*tcdB*) and regulatory genes (*tcdR*, *spoA*, *codY*, *ccpA*) were analysed by qRT-PCR using ChamQ Universal SYBR qPCR Master Mix Vazyme (Vazyme, Nanjing, China) following the manufacturer’s instructions [27]. All gene expression levels were normalized to the housekeeping gene 16S rRNA to account for variations in RNA input and reverse transcription efficiency. The reference *C. difficile* strain ATCC 43255 was used as a calibrator control for comparative analysis. Relative gene expression data were calculated using the 2^(-ΔΔCt) method, and fold changes were expressed relative to the control strain.

### Cytotoxicity assays and motility assays

The cytotoxicity assay was performed as described previously [28]. In brief, Vero cells were seeded in 96-well plates and incubated for 24 h. *C. difficile* strains were cultured anaerobically for 24 h. The collected supernatants were then filtered through 0.22 µm membranes. Serial ten-fold dilutions of each filtered supernatant were prepared in cell culture medium, and 20 µl of each dilution was added to the Vero cell monolayers. Following overnight incubation at 37 °C, Vero cells were examined under light microscopy. The endpoint titres were defined as the highest dilution that induced 50% cytopathic effect (CPE) in the cell population. The motility assays of *C. difficile* were performed as described previously [29]. *C. difficile* strains were cultured in BHI medium at 37 °C for 12 h to obtain overnight cultures. Subsequently, 2 µl of each overnight culture was carefully inoculated by puncturing the centre of BHI plates containing 0.5% agar. The inoculated plates were then incubated anaerobically at 37 °C for 3 days to allow bacterial spreading. Following incubation, the diameter of each bacterial colony was measured to assess motility.

### Statistical analysis

Statistical analysis was performed using GraphPad Prism (v9.0.0) and SPSS version 27.0. Two-way ANOVA, one-way ANOVA and the Kruskal–Wallis test were used to analyse data from biofilm formation, virulence gene expression, cytotoxicity and motility assays. Data are presented as mean±sem from three independent experiments. Statistical analysis of clinical data was performed using the Chi-square test, continuity-corrected Chi-square test or Fisher’s exact test. Statistical significance was set at *P*<0.05.

## Results

### Patients’ description

In this study, 100 *C. difficile* isolates were obtained, comprising 61 from symptomatic CDI patients and 39 from asymptomatic carriers. The demographic and clinical characteristics of the patients are summarized in Table 1. Among all participants, 23 (23%) were outpatients and 77 (77%) were hospitalized. The mean age was 6 years. The most common symptoms were abdominal pain (29%) and haematochezia (24%). Prior antibiotic exposure was identified in 61 (61%) patients, and 19 (19%) had a history of proton pump inhibitor use. Of the 100 patients, 79 (79%) experienced an initial episode of CDI, while 21 (21%) had recurrent infections. All asymptomatic patients were from the inpatient ward, and their number exceeded that of the symptomatic group (62.3% vs. 100%, *P*<0.001). Additionally, symptomatic patients were more prone to haematochezia (36.1% vs. 5.1%, *P*<0.001), while fever was less common in symptomatic patients compared to asymptomatic patients (9.8% vs. 43.6%, *P*<0.001). There were no statistically significant differences in other clinical characteristics between the two groups (*P*>0.05).

**Table 1.** Characteristics of all paediatric patients

Variable	Infection group (*n*=61)	Colonization group (*n*=39)	Total (*n*=100)	*P* value
Gender, male, *n* (%)	32 (52.5)	24 (61.5)	56	0.372
Age (year, median, range)	5.6 (4.6, 14.4)	6.4 (5.0, 16.5)	6	0.734
Community-acquired CDI, *n* (%)	50 (82.0)	33 (84.6)	83	0.731
Hospital-acquired CDI, *n* (%)	11 (18.0)	6 (15.4)	17	0.731
Outpatients, *n* (%)	23 (37.7)	0 (0.0)	23	<0.001
Inpatients, *n* (%)	38 (62.3)	39 (100.0)	77	<0.001
Exposure history, *n* (%)				
Antibiotics	35 (57.4)	26 (66.7)	61	0.353
Proton pump inhibitor	11 (18.0)	8 (20.5)	19	0.758
Prior CDI episode, *n* (%)	16 (26.2)	5 (12.8)	21	0.108
Comorbidity, *n* (%)				
Inflammatory bowel disease	6 (9.8)	5 (12.8)	11	0.891
Immunodeficiency	3 (4.9)	1 (2.6)	4	0.950
Symptoms, *n* (%)				
Fever	6 (9.8)	17 (43.6)	23	<0.001
Vomit	8 (13.1)	8 (20.5)	16	0.325
Abdominal pain	18 (29.5)	11 (28.2)	29	0.889
Haematochezia	22 (36.1)	2 (5.1)	24	<0.001
Pseudomembrane	5 (8.2)	0 (0.0)	5	0.173

### MLST and phylogenetic structure

**Fig. 1.**
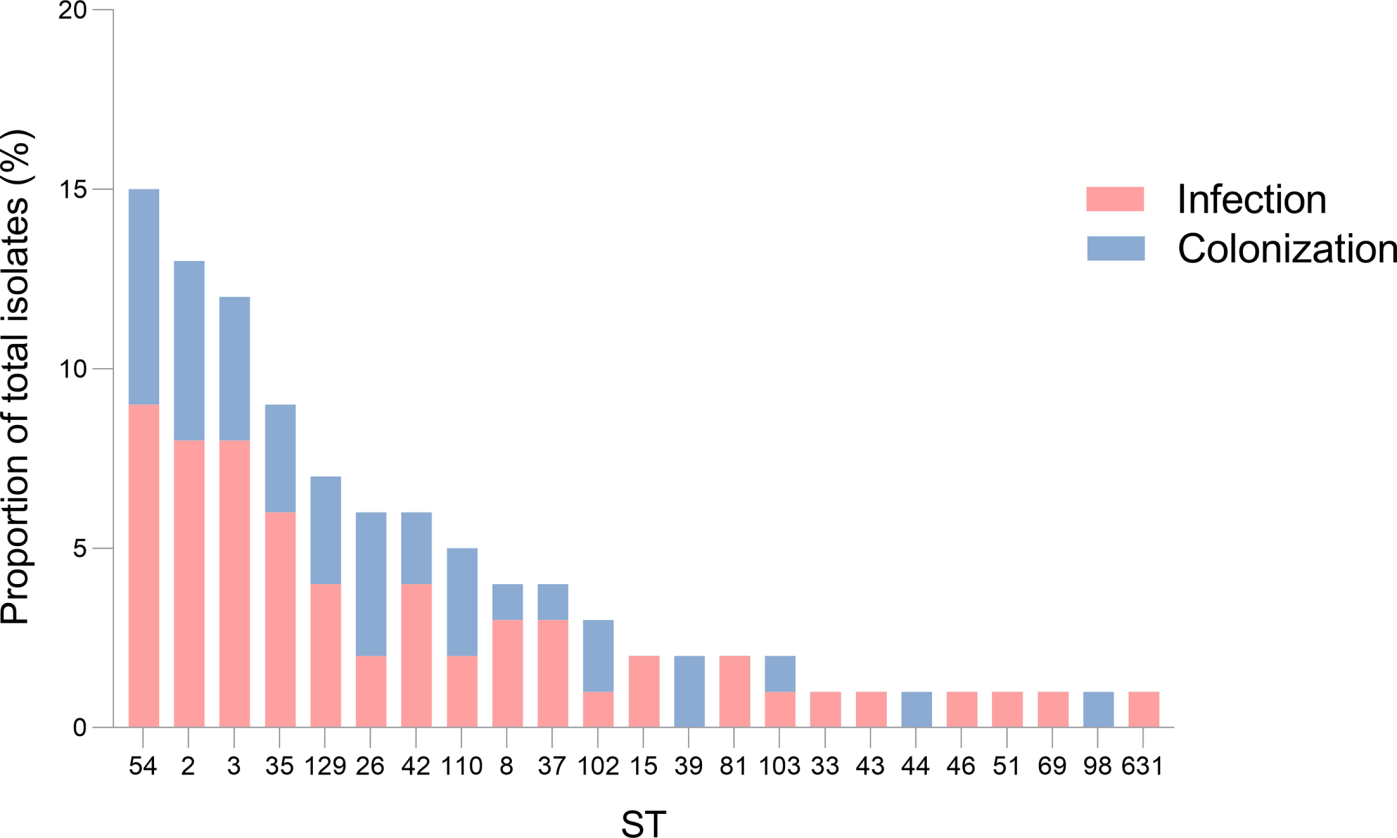
Bar chart showing the number of *C. difficile* isolates identified for each ST. Bar colours represent the source of the isolates – either from patients with CDI or from asymptomatic carriers. Red indicates isolates from CDI cases, while blue indicates isolates from asymptomatic carriers.

**Fig. 2.**
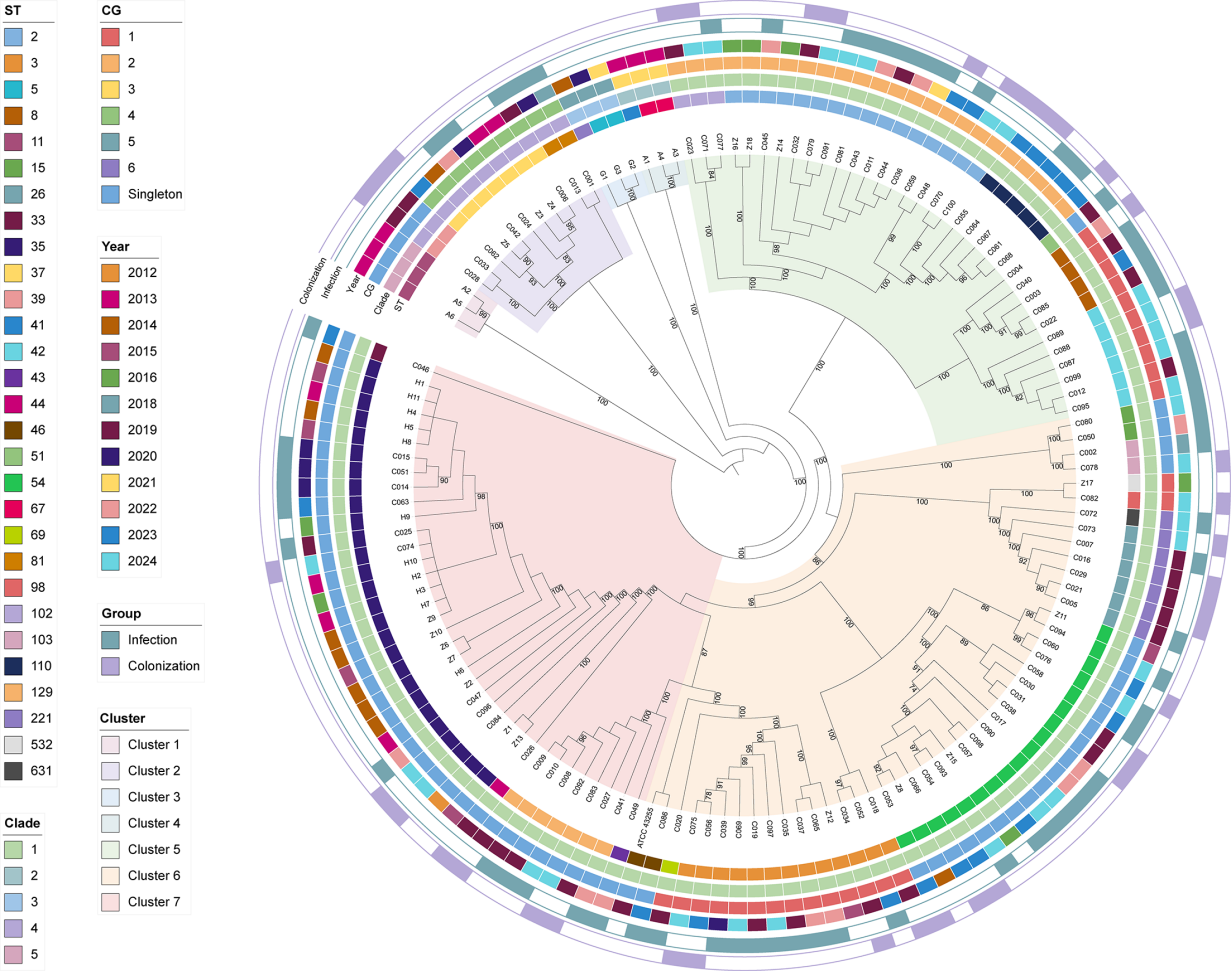
Phylogenetic analysis based on cgSNPs of *C. difficile*. ML phylogenetic tree of 138 *C. difficile* strains, including 100 isolates sequenced in this study and 38 publicly available genomes from NCBI database (Table S4). cgSNPs were used for tree construction, with the chromosome of reference strain ATCC 43255 as the reference genome. Tree branches are coloured according to MLSTs. From the innermost to the outermost ring, metadata annotations include ST, clade, CG, year of isolation, infection group (CDI) and colonization group (asymptomatic carriers).

A total of 23 sequence types (STs) were identified among the *C. difficile* isolates (Fig. 1, Table S1, available in the online Supplementary Material), with ST54 being the most prevalent (15%), followed by ST2 (13%) and ST3 (12%). Among the 61 isolates from symptomatic CDI patients, 20 distinct STs were identified. ST54 was the most prevalent (14.8%), followed by ST2 and ST3, each accounting for 13.1% of the isolates. In contrast, the 39 isolates from asymptomatic carriers were classified into 15 STs, with ST54 again being the most common (15.4%). Phylogenetic analysis based on cgSNPs revealed that the 100 *C. difficile* isolates clustered into two distinct major clades: Clade 1 (92%) and Clade 4 (8%) (Fig. 2). The 23 identified STs were subsequently classified into 6 CGs and 12 singletons. While isolates within the same CG typically demonstrated greater phylogenetic relatedness, they did not consistently cluster within identical phylogenetic branches.

### Genomic features and specific genes of *C. difficile* strains

**Fig. 3.**
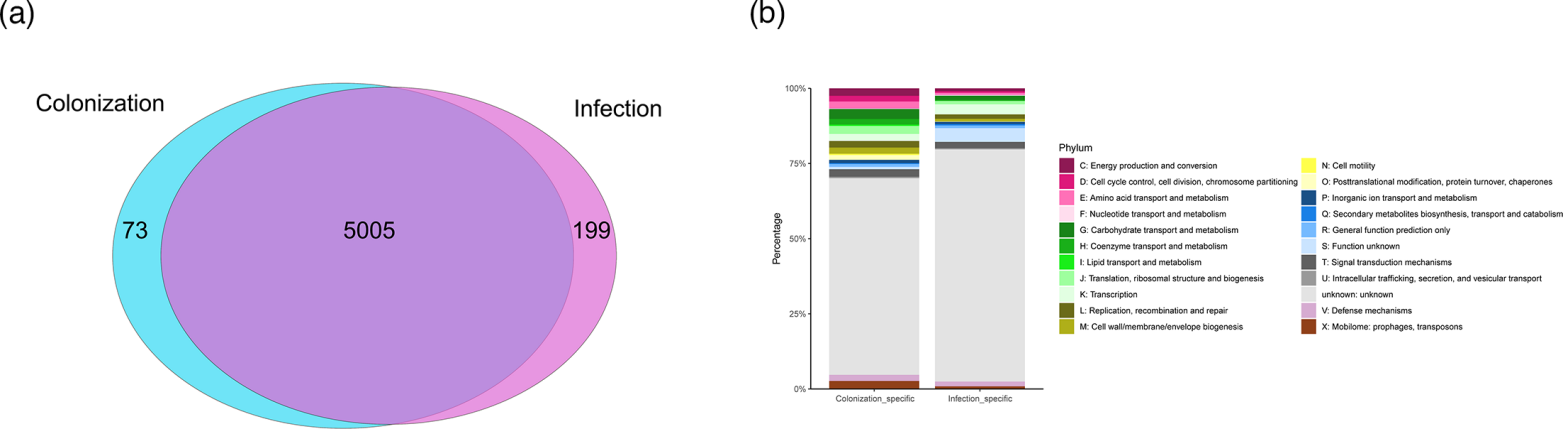
Distribution characteristics of orthologous genes in colonization and infection groups. (**a**) Venn diagram showing the number of shared and unique orthologous genes between colonization and infection groups. (**b**) Functional classification of group-specific genes based on the COG database, illustrating the COG category distribution of unique genes in the colonization and infection groups, respectively.

After quality control, a total of 1,557 Gbp of clean sequence data were obtained, with an average of 1.5 Gbp per sample (range, 0.743–7.043 Gbp). The average G+C content was 28.6 mol% (range, 28.1–29.3 mol%). *De novo* assembly of the reads yielded an average of 71 scaffolds (range, 28–994), with a mean N50 of 0.2 Gbp (range, 8,937–0.7 Gbp) (Table S2). Whole-genome analysis of *C. difficile* isolates identified a total of 379,168 genes, of which 377,993 (99.7%) were clustered into 5,277 orthologous gene families. Of these gene families, 5,005 (95.8%) were shared between the colonization and infection groups, indicating substantial genomic similarity between the two cohorts. In contrast, only 73 gene families were exclusive to the colonization group, while 199 were unique to the infection group (Fig. 3a). In total, the infection group harboured 808 unique genes, whereas the colonization group contained 639 unique genes.

To elucidate the functional characteristics of these group-specific genes, representative genes from each unique gene family were functionally annotated using the COG database (Fig. 3b). Functional analysis revealed that the majority of unique genes in both groups were uncharacterized. Specifically, 65.4% of genes in the colonization group and 77.2% in the infection group lacked definitive functional annotations. Among the functionally annotated genes, carbohydrate transport and metabolism represented the most enriched COG category in the colonization group. Conversely, the higher proportion of uncharacterized genes in the infection group suggests the presence of novel genetic elements that may contribute to *C. difficile* pathogenesis.

### Virulence, resistance and QS gene distribution

**Fig. 4.**
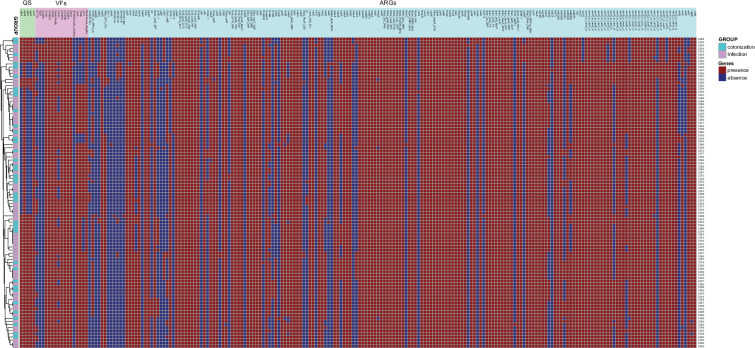
Distribution of QS genes, VF genes and ARGs in *C. difficile* isolates. A heatmap illustrates the presence or absence of specific genes across all isolates, including QS genes (*agrA*, *agrB*, *agrC*, *agrD*, *luxS*), VF genes identified via the VFDB and ARGs predicted using the CARD database.

Among exoenzymes, all isolates carried the *zmp1* and *cwp84* genes, whereas only one isolate (1%) harboured the streptococcal enolase gene. For adherence-related factors, all strains were positive for *CD0873*, *CD2831*, *CD3246*, *CbpA*, *FbpA*/*Fbp68* and *GroEL*. In contrast, *cwp66* and *SlpA* were detected in only 6% of isolates (*n*=6). Analysis of toxin-encoding genes revealed that 88 isolates (88%) were toxigenic, comprising 71 (80.7%) of the A^+^B^+^CDT^+^ genotype, 11 (12.5%) of the A^+^B^+^CDT^−^ genotype and 6 (6.8%) of the A^−^B^+^CDT^−^ genotype. The remaining 12 *C. difficile* strains exhibited the A^−^B^−^CDT^−^ genotype (Fig. 4). No significant difference in the proportion of toxigenic isolates was observed between the infection and colonization groups.

Analysis identified 196 antimicrobial resistance (AMR)-associated genes across the 100 *C. difficile* isolates, of which 126 genes (64.3%) were present in all isolates. Notably, several clinically significant resistance genes were detected, including *nimB-Cd* and *rpoB*, both of which are associated with treatment failure. Among the 70 non-ubiquitous AMR genes, 24 (12.2%) demonstrated carriage rates exceeding 50%, while the remaining 46 genes (23.5%) exhibited lower prevalence, with most detected in fewer than 10% of isolates. Notable resistance determinants included tetracycline resistance genes *tet(M)* (43%), *tetA(P)* (16%) and *tet(O)* (1%), as well as the *β*-lactam resistance gene *blaCDD-2* (26%). Notably, all isolates lacking *blaCDD-1* carried *blaCDD-2*, suggesting potential evolutionary divergence of *blaCDD-2* from *blaCDD-1* through genetic variation. Comparative analysis of AMR gene profiles revealed no statistically significant differences between the infection and colonization groups.

*luxS* and *agr1* were universally present (100%). The *agr2* locus was classified into two types, *agr2R* and *agr2M* (Table S3) [21]. In total, 46 (46%) isolates possessed *agr2R*, 8 (8%) isolates possessed *agr2M* and 6 (6%) isolates carried *agr3*. In the infection group, 31 (50.8%) isolates carried *agr2R*, 5 (13.1%) carried *agr2M* and 5 (8.2%) carried *agr3*. In the colonization group, 15 (38.5%) isolates carried *agr2R*, 3 (7.7%) carried *agr2M* and 1 (2.6%) carried *agr3*. Statistical analysis indicated no significant differences in the distribution of agr types between the two groups (*P*>0.05).

### Toxin locus variability

**Fig. 5.**
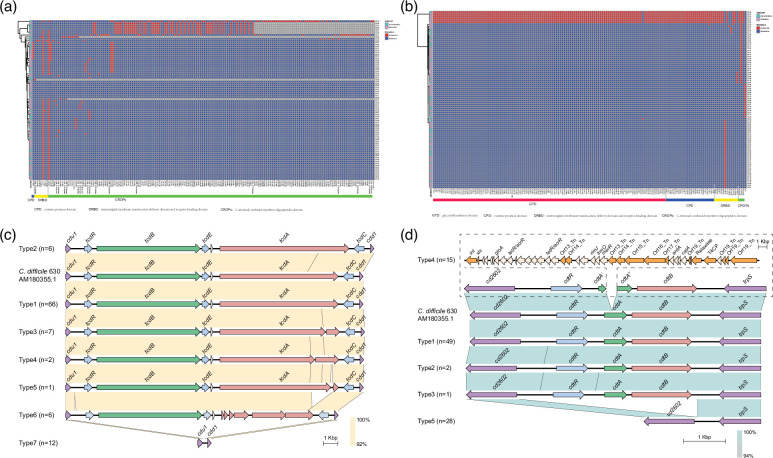
Analysis of TcdA, TcdB, PaLoc and CdtLoc regions in 100 *C. difficile* isolates. (**a**) and (**b**) Heatmaps showing mutation profiles of the TcdA and TcdB proteins. In the heatmaps, red indicates the presence of a mutation, blue represents no mutation and grey denotes the loss of downstream sites. The leftmost column indicates the sample grouping – colonization or infection. (c) Mutation sites are displayed in sequential order along the amino acid sequence. (**d**) Classification of isolates based on the genetic organization of the pathogenicity locus (PaLoc) and the binary toxin locus (CdtLoc).

Among the 82 *tcdA*-positive isolates, 73 (89%) harboured mutations, with similar rates observed in the infection group (86.3%) and colonization group (93.6%) (Fig. 5a). Similarly, 71 of 88 *tcdB*-positive isolates (80.7%) carried mutations, with comparable prevalence between the infection group (82.1%) and colonization group (78.1%) (Fig. 5b). The 65 strains covered only 12 out of the 155 mutation site types (7.7%). Notably, mutational diversity was significantly concentrated in a small subset of isolates (*P*<0.001); six isolates exhibited extensive multilocus mutation patterns, collectively accounting for 150 of the 155 total mutation site types (96.8%). Only one of these highly mutated isolates belonged to the colonization group, suggesting a predisposition for extensive *tcdB* variation in infection-associated strains. The PaLoc pathogenicity locus analysis classified the 100 isolates into seven distinct types (Fig. 5c). Importantly, 88 isolates harboured a PaLoc inserted between the *cdu1* and *cdd1* genes, while 12 isolates lacked the PaLoc entirely (Type 7). Type 1 PaLocs, which closely resembled the reference strain *C. difficile* 630, predominated in the collection. Type 2 variants exhibited tandem duplications at the 3′ end of *tcdA*, resulting in extended gene length compared to Type 1. Types 3, 4 and 5 each contained premature stop codons within *tcdA*, effectively dividing the locus into two distinct segments in our schematic representation. Type 6 PaLocs were characterized by multiple internal premature stop codons in *tcdA* and additional small deletions at the 3′ end, indicative of homologous recombination events within the gene.

CdtLoc analysis revealed five distinct structural types (Fig. 5d). In Types 1–4, the CdtLoc was consistently inserted between *cdtS* and *trpS*, the flanking genes of the cdtA–cdtB operon. In contrast, Type 5 isolates lacked the CdtLoc entirely. Type 4 represents a unique variant characterized by a 33,858 bp insertion within the *cdtA* coding sequence. Functional annotation of this insertion revealed integrase (int) and excisionase (xis) genes, consistent with an integrative and conjugative element (ICE) as confirmed by ICEfinder analysis. This ICE insertion disrupts *cdtA* functionality, likely abolishing binary toxin production and potentially attenuating virulence in affected strains.

### CF differences

**Fig. 6.**
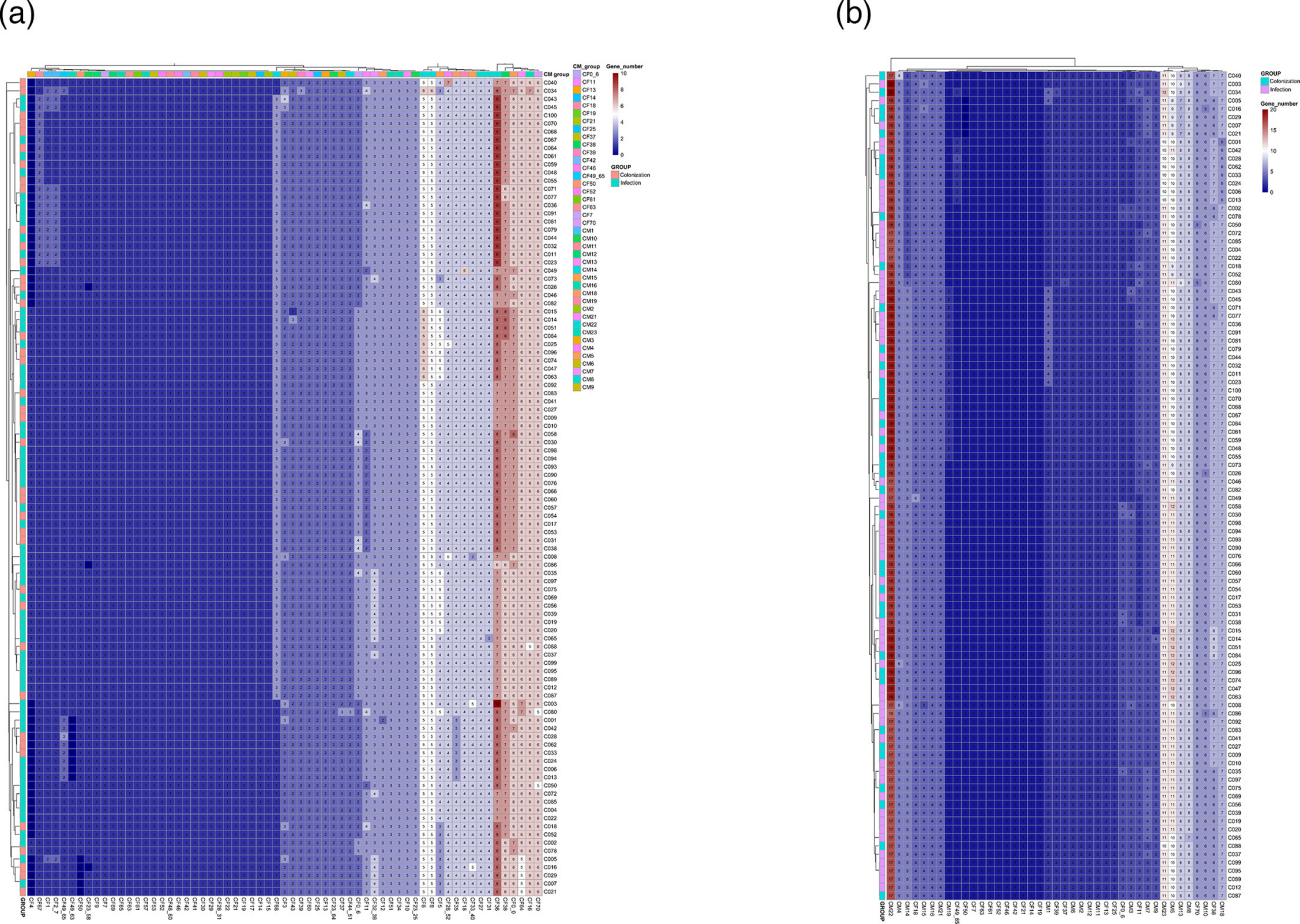
Distribution of CFs and CMs in *C. difficile* isolates. (**a**) and (**b**) Phylogenetic profiles of 63 CFs and 41 CMs across 100 *C. difficile* strains. In the heatmaps, the numbers within each cell indicate the count of CFs or CMs detected in each isolate.

A total of 63 distinct CF types were identified, with individual isolates harbouring 60–63 CF types. Copy numbers per CF type ranged from 0 to 10 (Fig. 6a). CF4 demonstrated the lowest prevalence, with 50% of isolates carrying a single copy and 50% lacking this factor entirely. Conversely, CF36 exhibited the highest copy numbers, with ≥7 copies present in 99% of isolates. No significant correlation was observed between CF copy numbers and colonization module (CM) grouping (Fig. 6b). Notably, the highest CF36 copy numbers were observed in CM22. Comparative analysis between infection and colonization groups revealed broadly similar mean CF copy numbers across all CF types. The largest mean differences were observed for CF5 (4.2 vs. 4) and CF36 (7.8 vs. 8.0), but the differences were not statistically significant.

### Biofilm formation, cytotoxicity, toxin expression and motility assay

**Fig. 7.**
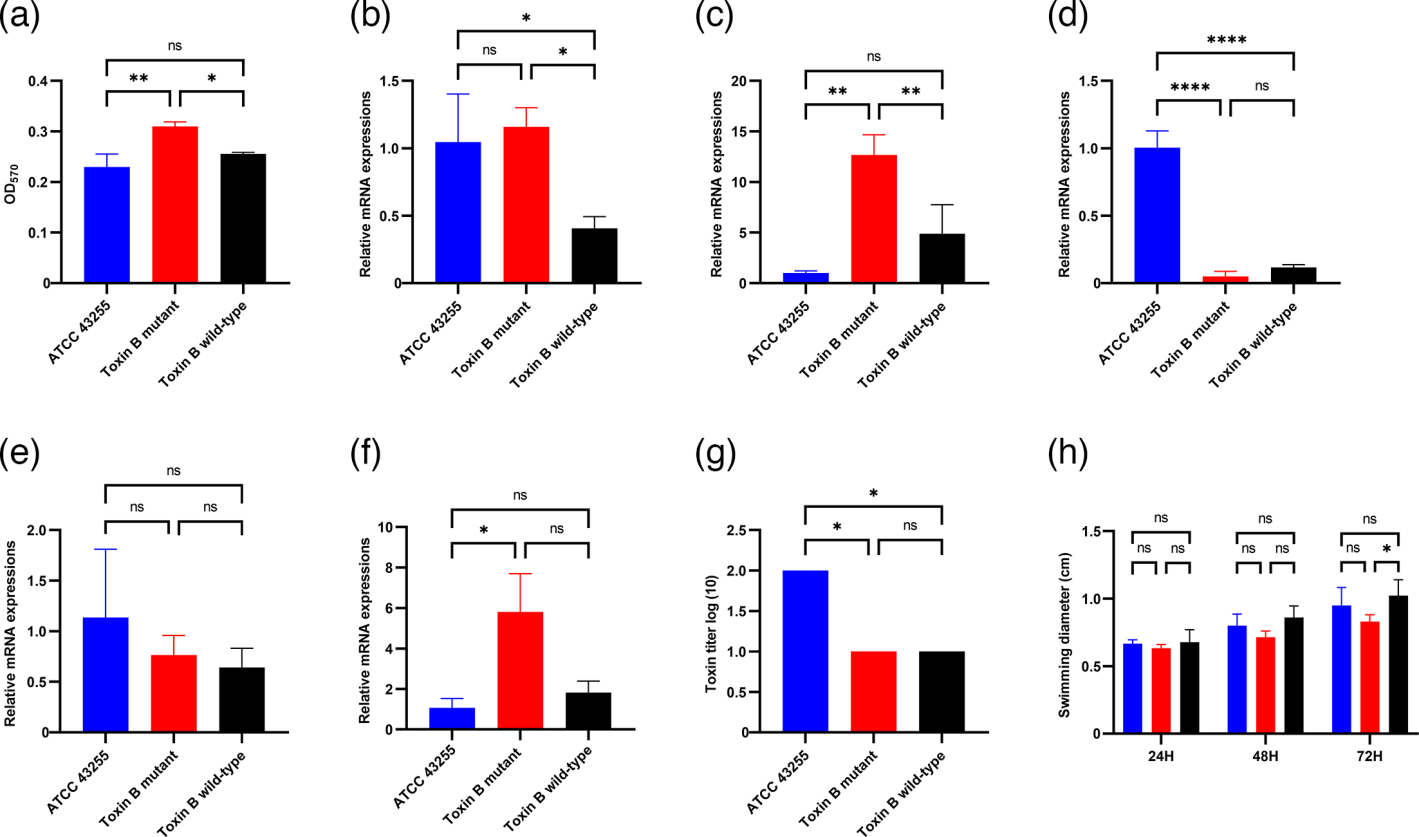
Comparative analysis of biofilm formation, gene expression, cytotoxicity and motility between *C. difficile* strains with and without toxin B gene mutations. (**a**) Biofilm formation capacity measured by crystal violet staining. OD at 570 nm represents biofilm biomass for *C. difficile* ATCC 43255, six toxin B gene mutant strains and three wild-type toxin B strains. (**b)–(f**) Relative gene expression levels of (b) *tcdB*, (c) *spoA*, (d) *tcdR*, (e) *ccpA* and (f) *codY* were determined by qRT-PCR. Expression levels were normalized to 16S rRNA and presented as fold changes relative to ATCC 43255. (**g**) Cytotoxic effects on Vero cells. (**h**) Motility assessment showing swimming zones on semi-solid agar medium after 24, 48 and 72 h anaerobic incubation at 37 °C. Zone diameters were measured and compared among strain groups. Blue represents ATCC 43255, red indicates toxin B gene mutant strains and black denotes wild-type toxin B strains. Data represent mean±sd from three independent experiments performed. *P<0.05, **P<0.01, ***P<0.001,****P<0.0001; ns, not significant.

We compared biofilm formation capabilities among six *tcdB* mutant strains, three wild-type *tcdB* strains and the reference strain ATCC 43255 (Fig. 7a). The six *tcdB* mutant strains comprised two ST81 isolates and four ST37 isolates, whereas the three wild-type (non-mutant) *tcdB* strains were all ST54 isolates. The reference strain ATCC 43255 belonged to ST46. Our results show that the *tcdB* mutant strains exhibited the highest biofilm formation, while ATCC 43255 showed the lowest biofilm formation after 3 days of incubation.

The relative mRNA expression level of *tcdB* was marginally elevated in ATCC 43255 and *tcdB* mutant strains relative to *tcdB* wild-type strains. Notably, *spoA* transcriptional expression in *tcdB* mutant strains was significantly upregulated compared to the other experimental groups. In contrast, ATCC 43255 demonstrated the most pronounced expression of *tcdR* among all tested strains. Conversely, *ccpA* expression levels showed no statistically significant variation across the three experimental groups. Furthermore, *codY* expression was significantly upregulated in *tcdB* mutant strains compared to ATCC 43255, as demonstrated in Fig. 7(b–f) (*P*<0.05).

Cytotoxicity assays revealed that CPE were observed at a 1:100 dilution for ATCC 43255 bacterial supernatants, whereas CPE were only detected at a 1:10 dilution for the other two bacterial groups (Fig. 7g). Swimming motility assays revealed no significant differences among the three bacterial groups when assessed at 24, 48 and 72 h post-inoculation (Fig. 7h).

## Discussion

Over the past few decades, the incidence of CDI in children has shown a steady increase, imposing a substantial burden on paediatric healthcare systems [30]. A recent cross-sectional study conducted at a tertiary children’s hospital in Zhejiang Province, China, reported that 14.3% of acute gastroenteritis cases were solely attributable to CDI [31]. This finding underscores the emerging role of CDI as a significant contributor to gastrointestinal infections in paediatric populations. In parallel, asymptomatic colonization of *C. difficile* has become increasingly prevalent in children. Studies have shown that up to 25% of hospitalized paediatric patients [32] and over 30% of paediatric oncology or stem cell transplant patients may carry *C. difficile* without exhibiting clinical symptoms at admission [33]. Asymptomatic carriers of *C. difficile* are frequently overlooked, yet they can serve as reservoirs of infection by shedding spores that contribute to outbreaks and facilitate transmission in both community and healthcare settings [34]. Recent evidence indicates that the risk associated with *C. difficile* carriage varies significantly by age group. In adults, asymptomatic carriers have been shown to be at increased risk of developing symptomatic CDI compared to non-carriers [35,36]. However, the situation differs markedly in paediatric populations. There is limited research on the progression from asymptomatic carriage to symptomatic infection in children. Current evidence suggests that children with inflammatory bowel disease who carry * C. difficile* are at a higher risk of developing CDI [37]. Infants and young children under 2 years commonly harbour *C. difficile* asymptomatically, exceeding 40% within the first year of life. Despite this high carriage rate, symptoms rarely occur, and CDI is not considered in the absence of high-risk factors [30,38]. Therefore, identifying the distinguishing features between *C. difficile* colonization and infection in older children and adults is critical for accurate diagnosis and suitable management.

To further investigate the genetic relationships among *C. difficile* strains isolated from paediatric patients with CDI and asymptomatic colonization, we performed phylogenetic and core genome analyses. ST54, ST2 and ST3 emerged as the predominant genotypes. Consistent with our findings, a recent global study identified ST2, ST42 and ST8 as the predominant genotypes worldwide, while ST3, ST37, ST54 and ST35 were most prevalent in China [34]. Our findings also show striking similarity to a paediatric study from Southwest China, which identified ST3, ST35 and ST54 as predominant types, nearly identical to our ST54, ST2 and ST3 distribution [39]. These findings indicate significant geographic heterogeneity in the global distribution of *C. difficile* STs, whereas the dominant STs circulating among paediatric populations in China appear comparatively conserved.

To elucidate genomic differences between the symptomatic and asymptomatic groups, group-specific genes were functionally annotated using the COG database. A substantial proportion of these genes were of unknown function, with notable enrichment in isolates from symptomatic patients. This observation is consistent with previous reports showing that certain genotypes, such as ST81 and ST37, harbour numerous uncharacterized genes, complicating comparative genomic analyses [40]. These findings suggest that the genomic divergence between symptomatic infection and asymptomatic colonization may be largely attributable to genes with currently unknown or poorly defined functions.

The study also investigated VF genes, ARGs and QS-associated genes. Notably, *slpA* and *cwp66*, two key genes involved in adhesion, were predominantly detected in *C. difficile* isolates from symptomatic patients, suggesting a potential role in promoting colonization and disease onset. Although each faecal specimen contains toxin-producing *C. difficile*, non-toxigenic *C. difficile* may also be present as co-infecting strains. Since only one *C. difficile* isolate is obtained from each faecal specimen, some of the isolates may be non-toxigenic. These strains may indeed function as normal gut commensals rather than pathogens. Non-toxigenic strains competitively exclude toxigenic strains through niche occupation and resource competition, potentially providing a protective effect against CDI. Animal studies have demonstrated that non-toxigenic *C. difficile* can prevent toxigenic CDI [41,42]. In addition, several ARGs were identified, including *nimB-Cd*, which confers resistance to nitroimidazoles, and mutations in *rpoB* potentially associated with resistance to fidaxomicin and rifaximin. These resistance determinants may contribute to treatment failure and persistence under antimicrobial pressure. *C. difficile* employs two QS systems – LuxS/autoinducer-2 (AI-2) and the accessory gene regulator (Agr) system – to modulate the expression of VFs [20,43]. In our study, QS-associated genes showed a numerically higher detection rate in isolates from symptomatic patients compared to those from asymptomatic carriers, although this difference did not reach statistical significance. While this preliminary observation warrants further investigation, the current data do not permit definitive conclusions regarding the role of QS systems in *C. difficile* virulence.

Furthermore, we analysed the mutational landscape and genetic diversity of toxin-encoding genes among the *C. difficile* isolates. Notably, frequent multisite mutations within the *tcdB* gene were observed in ST37 and ST81 strains, the majority of which were isolated from symptomatic patients. This pattern suggests a possible association between *tcdB* polymorphisms and clinical disease severity. *In vitro* analysis demonstrated that ST81 and ST37 toxin B variants displayed increased biofilm production and upregulated *tcdB* and sporulation gene expression relative to clinical strains lacking these variants. ST37 has been linked to clinical manifestations comparable in severity to those caused by the hypervirulent ST1 strain [27,44]. ST81 isolates demonstrate enhanced transmissibility, robust environmental persistence and increased colonization potential, characterized by high sporulation efficiency and modestly elevated motility [40]. These features underscore the need for heightened surveillance and early identification of ST37 and ST81 strains in clinical settings. In this study, we employed a conserved genetic module-based approach to identify potential determinants of intestinal colonization [25]. To our knowledge, this is the first investigation to compare colonization-associated genetic features between paediatric *C. difficile* strains isolated from symptomatic infections and asymptomatic carriage. Among the CFs analysed, CF36 and CF5 exhibited the most pronounced differences between symptomatic and asymptomatic *C. difficile* strains. CF36 encodes components of the *β*-glucoside-specific phosphotransferase system (PTS), including PTS enzyme II and regulatory elements such as BglG kinase and BglG phosphatase, which modulate *β*-glucoside utilization operons. Carbohydrates serve as a crucial energy source for *C. difficile*. It can metabolize various simple sugars through the PTS, including fructose, glucose, mannose, mannitol, melibiose, sorbitol, trehalose, cellobiose and mucin-derived glycans [45]. It has been demonstrated that glucose and fructose promote sporulation and the ability to colonize in *C. difficile* [46]. PTS may enhance *C. difficile*’s ability to exploit host- or microbiota-derived carbohydrates, thereby promoting intestinal colonization. In contrast, CF5 displayed a distinct distribution pattern across isolates, suggesting a potentially complementary role in colonization or persistence. These differences in CF36 and CF5 likely reflect varying metabolic capabilities and environmental adaptation strategies that contribute to colonization dynamics, particularly in symptomatic paediatric patients.

This study has several limitations. First, it was conducted at a single tertiary paediatric centre, which may limit the generalizability of the findings. Second, only one *C. difficile* isolate per patient was analysed, which may have overlooked intra-host strain diversity and the presence of co-colonizing strains with differing virulence potential. Third, the lack of longitudinal follow-up data precluded the evaluation of clinical progression among asymptomatic carriers, limiting insights into the temporal dynamics of colonization and disease onset. Fourth, we employed whole-genome next-generation sequencing. However, due to the limitations of short-read sequencing technology, certain genomic regions or specific genes may not be fully captured, which could result in the omission of some genetic elements.

## Conclusion

This study is the first to comprehensively characterize and compare the genomic features and virulence-associated factors of * C. difficile* isolates from paediatric patients with symptomatic infection and asymptomatic colonization. Our findings reveal distinct genetic and functional profiles between the two groups, including differences in STs, CG distribution, CFs and virulence gene content. Notably, a substantial proportion of group-specific genes were of unknown function, highlighting the complexity and uniqueness of the *C. difficile* genome in paediatric populations. These results enhance our understanding of the genomic basis underlying colonization vs. infection and may inform future diagnostic, therapeutic and preventive strategies for paediatric CDI.

## Supplementary material

10.1099/mgen.0.001610 Uncited Supplementary Material 1.

## Abbreviations

AMR: antimicrobial resistance
ARGs: antimicrobial resistance genes
BHI: brain heart infusion
CDI: *Clostridioides difficile* infection
CDT: *Clostridioides difficile* binary toxin
CFs: colonization factors
CG: clonal group
cgSNPs: core genome SNPs
CM: colonization module
COG: Clusters of Orthologous Groups
CPE: cytopathic effect
ICE: integrative and conjugative element
ML: maximum-likelihood
MLST: multilocus sequence typing
NAAT: nucleic acid amplification test
PTS: phosphotransferase system
qRT-PCR: quantitative real-time PCR
QS: quorum sensing
STs: sequence types
VFs: virulence factors
WGS: whole-genome sequencing
